# Assessing cellular and circulating miRNA recovery: the impact of the RNA isolation method and the quantity of input material

**DOI:** 10.1038/srep19529

**Published:** 2016-01-20

**Authors:** Victoria El-Khoury, Sandrine Pierson, Tony Kaoma, François Bernardin, Guy Berchem

**Affiliations:** 1 Department of Oncology, Luxembourg Institute of Health (LIH), 84 Val Fleuri, L-1526 Luxembourg, Luxembourg; 2 Centre Hospitalier de Luxembourg, 4 rue Barblé, L-1210 Luxembourg, Luxembourg

## Abstract

MicroRNAs (miRNAs) have emerged as promising cancer biomarkers. However, exploiting their informative potential requires careful optimization of their detection. Here, we compared the efficiency of commonly used RNA extraction kits in miRNA recovery from cells, plasma and urine/plasma-derived exosomes, using single-gene RT-qPCR and miRNA profiling. We used increasing amounts of starting material to investigate the impact of the input material size on miRNA extraction. We showed that miRNA recovery was largely influenced by the isolation method and by the amount of input material. In particular, the miRCURY™ kit provided highly pure RNA. However, its columns poorly recovered miRNAs from limiting amounts of cells and plasma, and rapidly saturated by large RNA species and plasma components, thus impeding miRNA recovery from high input amounts. Overall, the miRNeasy^®^ kit permitted a better miRNA detection despite a less pure extracted RNA. Nevertheless, some miRNAs were preferentially or exclusively isolated by either of the methods. Trizol^®^ LS resulted in very low purity RNA which affected RT-qPCR efficiency. In general, miRCURY™ biofluids kit efficiently extracted miRNAs from plasma. A careful selection of the RNA isolation method and the consideration of the type and size of input material are highly recommended to avoid biased results.

MicroRNAs (miRNAs) are small non-coding RNAs that downregulate gene expression through their binding to 3′UTR target mRNAs^1^,^2^.

Since their discovery in bodily fluids, inside membrane-vesicles (exosomes, microvesicles etc.) or bound to proteins and lipoproteins^3^,^4^, and due to their high stability and their informative power, circulating miRNAs have emerged as promising non-invasive cancer biomarkers^5^,^6^,^7^. However, their clinical significance might not be clearly demonstrated without an accurate optimization and standardization of the protocols used for miRNA assessment^1^,^8^.

Indeed, several inconsistencies have been observed among the studies and dissimilar results could be explained in part by differences in methodological parameters^9^,^10^,^11^,^12^,^13^. In this context, the various RNA extraction methods, amounts and types of input material used in miRNA analyses appear to contribute significantly to the poor overlap of results among different studies^1^,^14^.

RNA extraction methods can be roughly classified into three main groups: 1) the phenol-based techniques that rely on the use of organic solvents, phase separation, and recovery of RNA by precipitation, 2) the combined phenol and column-based techniques that utilize phenol and chloroform to separate RNA from other constituents, and a column for RNA adsorption, 3) the column-based techniques that use a phenol-free lysis buffer and a column for RNA recovery^8^,^15^.

Several efforts have been made towards the comparison and the optimization of extraction methods for miRNA^11^,^14^,^15^,^16^,^17^,^18^,^19^, driven by the discovery of miRNAs as potential candidate biomarkers^5^,^7^,^20^. Still, lots of interlaboratory differences in results exist. In this regard, Tan GW *et al*.^19^ found that miRCURY™ biofluids and miRNeasy^®^ Serum/Plasma kits were comparably efficient in recovering miRNA spike-in controls from plasma samples, whereas McAlexander M *et al*.^21^ suggested a slightly lower recovery by miRNeasy^®^ Serum/Plasma kit versus miRCURY™ biofluids kit. On a different note, Cheng L *et al*. demonstrated that the miRNeasy^®^ kit was slightly more efficient than the miRCURY™ kit in extracting miRNAs from urinary exosomes^12^. They also showed that, among six commercially available kits, the miRNeasy^®^ and the mirVana™ kits were the most performant ones for maximizing RNA extraction from urinary exosomes isolated by ultracentrifugation^12^. Conversely, a higher detection of mir-16 and let-7i from urinary exosomes was reported when using miRCURY™ instead of miRNeasy^®^^9^. The presence of high levels of contaminants in the phenol-based preparations has been suggested to account, at least partly, for the differences in miRNA recovery by the different methods^9^. It should be pointed out that the protocols are adapted by each research group which may also contribute to generating inconsistent results between these studies. Therefore, there is an urgent need of standardization and improvement of existing methods. This could be achieved when different extraction techniques are compared in the same study using several types of starting material (different states, densities and/or constituents) and variable quantities of input material; It would be then possible to accurately interpret the results and to better understand the incoherencies observed among different studies.

Here, we have compared commonly used RNA extraction methods for their efficiency in miRNA isolation from different sample types and varying amounts of input material. The assessment of miRNA recovery has been evaluated by capillary electrophoresis and by reverse transcription quantitative real-time polymerase chain reaction (RT-qPCR) through single gene examination and a broad miRNA profiling. In particular we chose to evaluate a phenol- (Trizol^®^ LS), two column- (miRCURY™ RNA Isolation and miRCURY™ biofluids kits) and a combined phenol- and column-based (miRNeasy^®^ Mini kit) RNA isolation techniques. Besides, we investigated the impact of the amount of starting material on the efficiency of miRNA extraction by using variable numbers of cells and different volumes of plasma. This is of particular importance since the use of “unlimited” quantity of material from cells in culture may not yield results that mirror analyses performed with limited-size clinical samples. Besides, some measurements are not feasible with clinical specimens; this is for example the case of plasma RNA quantification with NanoDrop spectrophotometer. In such situations, a fixed volume of input material rather than an equal quantity of RNA should be used^15^. Accordingly, we decided to perform this comparative study using both a fixed RNA volume and the same amount of RNA from cell lysates, in order to evaluate their impact on miRNA detection by each method. Finally, we have tested the performances of the RNA extraction methods with different sample types, using cells in culture, plasma, and extracellular vesicles derived from plasma and urine. To the best of our knowledge, such wide comparison has never been done before. It is also worth mentioning that Exiqon kits and isolation of exosomal miRNAs from plasma and urine have been poorly investigated in such comparative studies.

## Results

### Comparison of RNA yield and integrity

We first evaluated the efficiency of Trizol^®^ LS, miRNeasy^®^ and miRCURY™ kits in extracting total RNA from A549 samples containing either low (25 × 10^3^), medium (200 × 10^3^) or high (800 × 10^3^) cell numbers (25c, 200c and 800c, respectively).

Independently of cell density, miRNeasy^®^ and miRCURY™ kits yielded similar RNA quantities suggesting that they were equally efficient in total RNA extraction from cells in culture (Table 1).

When the starting cell number was high, Trizol^®^ LS extracted less RNA than miRNeasy^®^ and miRCURY™ (244.5 ± 90.4 ng/μL versus 339.5 ± 153.4 and 341.2 ± 184.4 ng/μL, respectively). However with medium and low cell numbers, the efficacy of Trizol^®^ LS seemed increased. This apparent improvement likely resulted from a contamination by residual phenol that induced an erroneous overestimation of RNA quantities, due to the absorbance peak at 270 nm (Supplementary Fig. S1). The electropherogram overlay of RNA extracts from 25c cells supports the low total RNA isolation efficiency of Trizol^®^ LS over the two other methods (Supplementary Fig. S2).

As expected, the contribution of MS2 to RNA amounts was more substantial at low and medium cell densities than at high cell densities (Table 1).

The RNA integrity was evaluated based on the RNA Integrity Number (RIN). All three extraction methods yielded high RIN values (mean values >9.1), indicating a high quality RNA. Importantly, the presence of MS2 decreased the RIN to 6.5 in the 25c samples. Nevertheless, this low RIN should not be interpreted as an RNA degradation, since it is due to MS2 itself that changes the RNA electropherogram profile (Supplementary Fig. S2),

### Comparison of RNA purity

RNA purity was assessed by measuring the absorbance at 260 nm, 280 nm and 230 nm as described in the Methods. As expected, when the number of starting material increased, the purity of the sample increased as well, due to a higher RNA absorbance peak at 260 nm, independently of the extraction method (Table 2). At medium and high cell densities, the purity of the samples, as assessed by A260/A280 ratio, was satisfactory for the three isolation techniques, albeit better with miRNeasy^®^ and miRCURY™ versus Trizol^®^ LS. At low cell density, the A260/A280 ratio was low for all extraction methods (A260/A280 <1.8). This ratio raised above 1.8 only after the addition of MS2 RNA, indicating that the low RNA concentrations obtained with 25 × 10^3^ cells, rather than a considerable protein contamination, were the main reason behind the low A260/A280 ratio.

The purity of the samples as assessed by the A260/A230 ratio was substantially different between the three isolation techniques. Whereas miRCURY™ kit yielded pure RNA at high cell density (A260/230 ≥ 1.95) and RNA with acceptable purity (A260/230 ≥1.6) at medium cell density, the A260/A230 was substantially low in Trizol^®^ LS and miRNeasy^®^ extracts for all starting cell numbers, indicating an important presence of contaminants that absorb at 230 nm (Table 2). Indeed, the presence of phenol is easily recognizable by the absorption peaks at ∼270 nm and at ∼230 nm in the samples extracted with Trizol^®^ LS and miRNeasy^®^ (Supplementary Fig. S1). The guanidinium thiocyanate may also account for the high absorbance at 230 nm. The spectral patterns of miRCURY™-based extraction displayed one single peak at 260 nm, typical of pure RNA (Supplementary Fig. S1). Importantly, the low A260/A230 ratio of the 25c samples extracted with miRCURY™ was likely related to the low absorbance at 260 nm that cannot balance the measurement at 230 nm, rather than a considerable presence of contaminants (the absorbance at 230 nm is negligible in these samples).

### Differential efficiency of RNA extraction methods in the detection of miRNAs by RT-qPCR

We investigated the performances of Trizol^®^ LS, miRNeasy^®^, and miRCURY™ kits in downstream RT-qPCR experiments for miRNA detection, using either a fixed volume of eluted RNA (previously diluted when indicated; see Methods for details) or a fixed amount of RNA (5 ng) as input for RT reaction.

**Figure 1:**
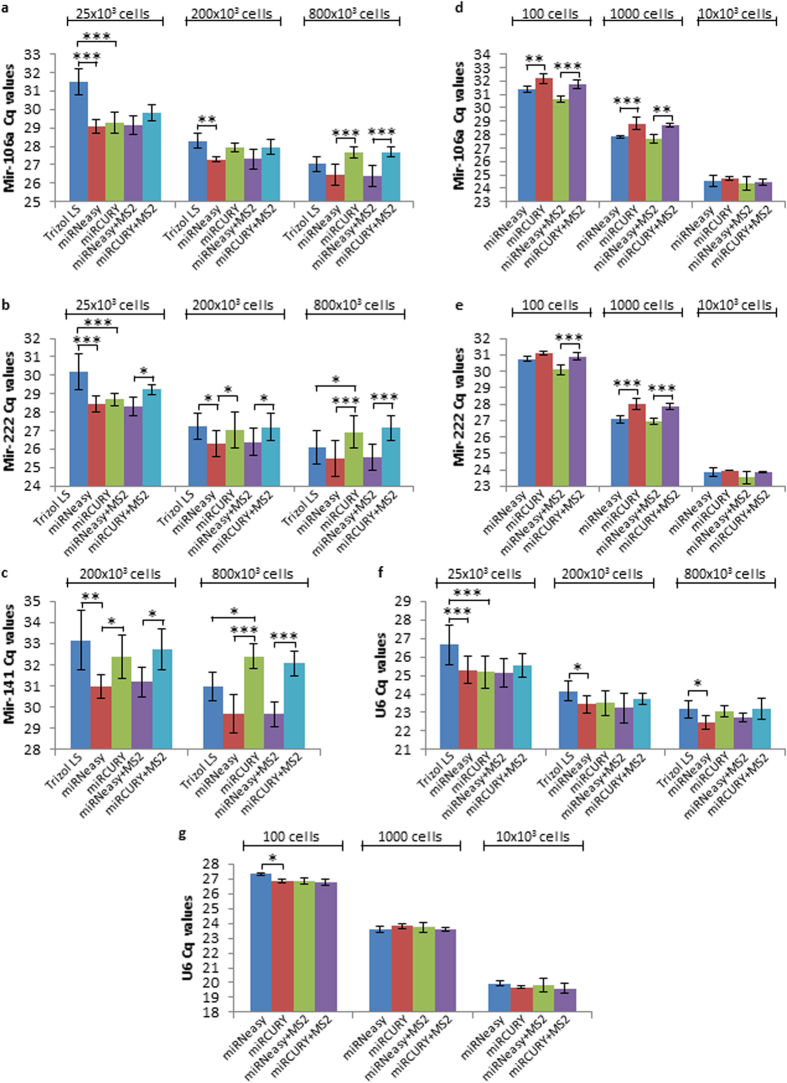
Efficiency of RNA extraction methods for miRNA detection by RT-qPCR in different cell density conditions, using fixed RNA volumes. RNA samples from (**a,b,c,f**) 25 × 10^3^, 200 × 10^3^ and 800 × 10^3^ A549 cells (n = 3) and (**d,e,g**) 100, 1000 and 10 × 10^3^ A549 cells (n = 3) were obtained by extraction with either Trizol^®^ LS, miRNeasy^®^, or miRCURY™, in the presence or absence of MS2 carrier. The results represent average Cq values obtained for (**a,d**) mir-106a, (**b,e**) mir-222, (**c**) mir-141 and (**f,g**) U6 snRNA. The detection of miRNA was performed by RT-qPCR using a fixed volume of RNA samples (see Methods for details). The mean values ± SD of 3 independent experiments are shown. *P <* 0.05* **P <* 0.01* ***P <* 0.001.*

With a fixed RNA volume and a low cell density, miRNeasy^®^ and miRCURY™ kits resulted in equal detection of mir-106a and mir-222 (similar Cq values), whereas Trizol^®^ LS extraction yielded significantly higher Cq values (*P < 0.001*) suggesting a lower recovery of miRNAs and/or a poor PCR efficiency due to phenol contamination (Fig. 1a,b). When the cell number is increased to 200 × 10^3^, miRNAs were detected at higher levels with miRNeasy^®^ versus Trizol^®^ LS and miRCURY™ (lower Cq values with miRNeasy) (Fig. 1a–c). At higher cell density, the Cq differences between miRNeasy^®^ and miRCURY™ increased (*P < 0.001*), suggesting a better detection of mir-106a, mir-222, and mir-141 with miRNeasy^®^. Whilst miRCURY™ outperformed Trizol^®^ LS at low cell density, Trizol^®^ LS permitted a better detection of miRNAs at high cell density, albeit less efficiently than miRNeasy^®^. The results obtained with MS2-supplemented conditions were comparable to those obtained without MS2 (mainly with medium and high cell numbers), for each of miRNeasy^®^ and miRCURY™.

Since clinical samples or sorted subpopulations may contain as few as one-hundred cells, we then compared the performances of miRNeasy^®^ and miRCURY™ kits using 100, 1000 and 10 × 10^3^ cells as starting material. Given the poor microRNA recovery observed with Trizol^®^ LS isolation from 25 × 10^3^ cells, this condition was not tested with fewer cell numbers. When the starting cell number is very low (100 and 1000 cells), miRNeasy^®^ kit permitted a better detection of mir-106a and mir-222 when compared to miRCURY™ kit, independently of MS2 supplementation (Fig. 1d,e). With 10 × 10^3^ cells, both methods proved equally efficient, similarly to what we already observed with 25 × 10^3^ cells as starting material. Since U6 small nuclear RNA (snRNA) is commonly used as a housekeeping gene to normalize miRNA expression in cells, we wished to know whether its detection is affected by the RNA extraction method, similarly to miRNAs. Our data show no or small, albeit statistically significant, fluctuations in U6 recovery (mainly a decreased recovery with Trizol^®^ LS) across the different techniques and with all the quantities of starting material investigated (Fig. 1f,g). Accordingly, when miRNA levels were normalized to U6 snRNA and displayed as fold change relative to miRNeasy^®^ condition, the results were comparable to the data obtained with the raw Cq averages, the fold changes being in general the highest with miRNeasy and miRNeasy + MS2 protocols (Supplementary Fig. S3).

**Figure 2:**
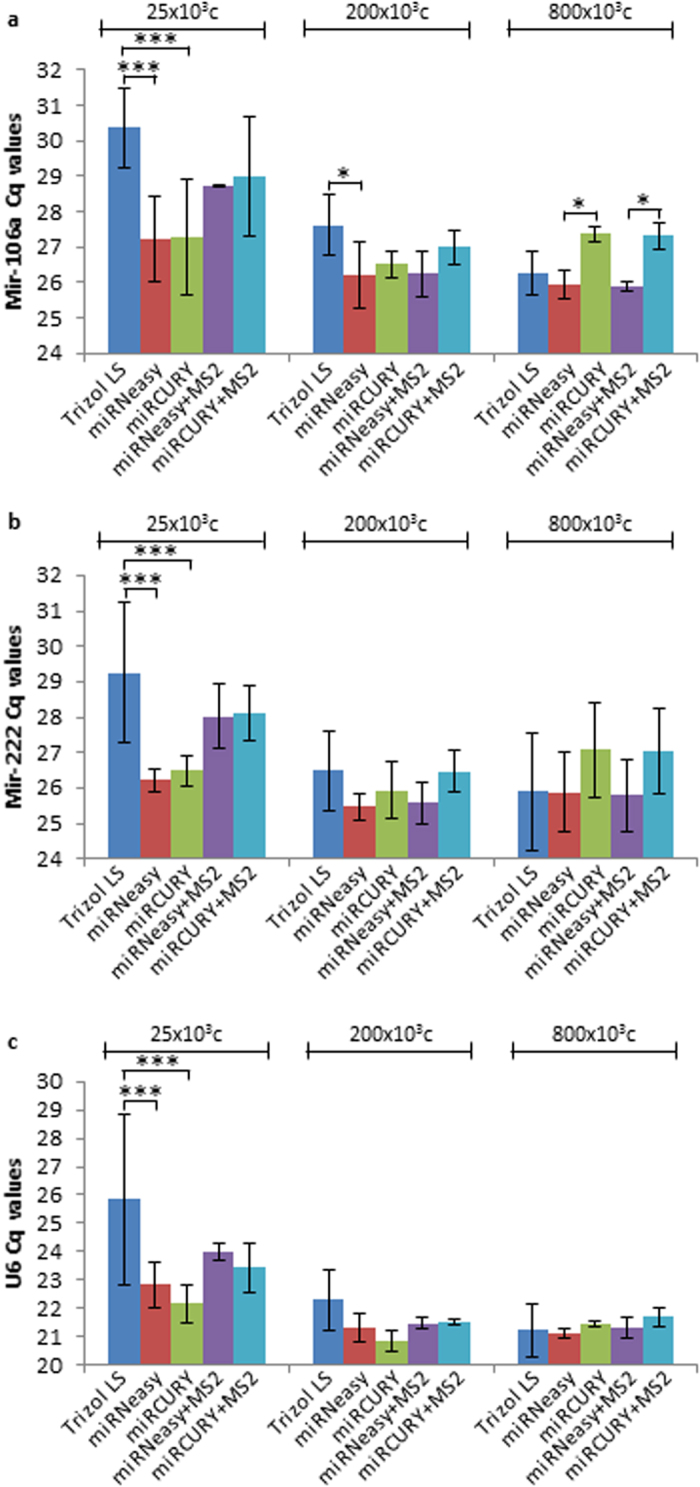
Efficiency of RNA extraction methods for miRNA detection by RT-qPCR in different cell density conditions, using a fixed RNA quantity. The results represent average Cq values obtained for (**a**) mir-106a, (**b**) mir-222 and (**c**) U6 snRNA. RNA samples from 25 × 10^3^, 200 × 10^3^ and 800 × 10^3^ A549 cells were obtained by extraction with either Trizol^®^ LS, miRNeasy^®^, or miRCURY™, in the presence or absence of MS2 carrier. The detection of miRNA was performed by RT-qPCR starting with 5 ng of total RNA/RT reaction. The mean values ± SD of 3 independent experiments are shown. *P <* 0.05* ***P <* 0.001.*

In order to analyze if differences in miRNA detection can also be observed when a fixed amount rather than a fixed volume of input RNA is used, RT-qPCR experiments were performed with 5 ng of RNA to detect the expression of mir-106a, mir-222 and U6 snRNA. As shown in Fig. 2a–c, the results were comparable to those obtained with a fixed RNA volume. Higher Cq values were obtained when MS2 was added to the low cell density conditions, certainly because MS2 accounted for a considerable amount of the final RNA in the eluate. The miRNA expression levels normalized to U6 snRNA and displayed as fold change relative to miRNeasy^®^ condition are shown in Supplementary Fig. S4. Overall, the fold change data suggest that miRNeasy^®^ isolation method is at least as efficient as miRCURY™ and Trizol^®^ LS. Some inconsistencies between the fold change data and the raw Cq values are seen and may result from (1) simultaneous variations of miRNAs and U6 Cq values, (2) the presence of MS2 RNA quantified together with cellular RNA, or (3) the presence of phenol in some samples which may alter the RNA quantification.

Altogether, these data demonstrate that miRNeasy^®^ isolation technique outperforms Trizol^®^ LS and miRCURY™ specifically in the detection of miRNAs by RT-qPCR. Our findings imply that miRNA recovery may be affected by the amount of input material.

### The recovery of miRNAs by miRCURY™ kit is altered when the starting cell number increases

To better understand how the amounts of starting material may influence miRNA yields, we analyzed the efficiency of miRNA recovery by each of the methods using increasing number of starting material and a fixed volume of eluted RNA.

When the cell number increases from 25 × 10^3^ to 200 × 10^3^, the Cq values should decrease and the theoretical mir-106a and mir-222 Cq difference should be of 1.38 (when considering the experimental procedure explained in Methods). The best Cq differences between these two cell densities were obtained with miRCURY™ extraction (ΔCq (25c–200c) = 1.342 ± 0.488 and 1.646 ± 0.633 for mir-106a and mir-222, respectively) (Table 3 and Supplementary Fig. S5), suggesting a very good PCR efficiency and an miRNA yield roughly proportional to cell number. Nevertheless, the Cq values obtained with miRNeasy^®^ were lower than those obtained with miRCURY™, indicating that miRNeasy^®^ extraction recovered a higher amount of mir-106a, mir-222 and mir-141. Whereas the Cq differences between the 200c and the 800c samples should be ideally of 2 for mir-141 and of 0.83 for mir-106a and mir-222 (see Methods for details), the miRCURY™ kit resulted in very low ΔCq (−0.009 ± 0.758, 0.272 ± 0.339 and 0.121 ± 0.208 for mir-141, mir-106a and mir-222, respectively), when compared to Trizol^®^ LS and miRNeasy^®^ (Table 3 and Supplementary Fig. S5), suggesting there was no or very small increase in miRNA recovery with 800 × 10^3^ cells versus 200 × 10^3^ cells. On the contrary, the experimental miRNeasy^®^ ΔCq were optimal, mainly for mir-106a and mir-222 (ΔCq = 0.832 ± 0.416 and 0.803 ± 0.269, respectively). Whereas the experimental ΔCq values were similar in the absence or presence of MS2 for miRNeasy^®^ protocol, the addition of MS2 worsened miRNA detection when using miRCURY™ kit with 25 × 10^3^ cells (Table 3).

These data suggest that, contrarily to miRNeasy^®^, the recovery of miRNAs with miRCURY™ decreases dramatically when increasing the amount of input material (albeit in the range recommended by the manufacturer). This observation implies a saturation of the column by RNA species or by protein-induced column clogging.

### Recovery of small RNAs including miRNAs by the different extraction methods

In order to investigate whether the differences observed in the gene-specific miRNA RT-qPCR data result from variations in the extraction efficiency of small RNAs, we used the Agilent Small RNA kit to quantify each of these fractions. This kit analyses small RNAs in the 6 to 150 nucleotide (nt)- size range and detects miRNAs in the 10 to 40-nt range. As shown in Table 4, the highest small and miRNA concentrations were obtained with the miRNeasy^®^ extraction, for both low and high starting cell numbers. At low cell density, the recovery of small RNAs was better with miRCURY™ than with Trizol^®^ LS. However, the concentration and percentage of miRNAs in this fraction were lower.

Whilst the abilities of miRNeasy^®^ and miRCURY™ to extract small RNAs were comparable at low cell density (small RNA concentration = 1.25 ng/μL and 1.15 ng/μL for miRNeasy^®^ and miRCURY™, respectively), at high cell density, the miRNeasy^®^ kit showed a better small RNA isolation ability (small RNA concentration = 29.62 ng/μL and 17.89 ng/μL for miRNeasy^®^ and miRCURY™, respectively). These differences are illustrated by the small RNA electropherogram overlays shown in Supplementary Fig. S6. The reduced ability of miRCURY™ kit to extract small RNA at high cell density likely accounted for the further drop of miRNA recovery (miRNA concentration = 1.17 ng/μL and 0.62 ng/μL for miRNeasy^®^ and miRCURY™, respectively).

These data demonstrate that miRNeasy^®^ kit permits a better isolation of small RNAs including miRNAs compared to Trizol^®^ LS and miRCURY™, and that small RNA extraction efficiency of miRCURY™ kit decreases when the starting cell number increases, ultimately resulting in reduced miRNA recovery.

### The miRCURY™ kit favours the isolation of large RNAs over miRNAs at high cell density

In an attempt to measure the small RNA fraction by an alternative method, we used the Qubit^®^ microRNA assay to quantify the amounts of small RNAs in the 800c samples. The concentrations of the so-called “small RNAs” were 51.6 ± 26.5 ng/μl, 101.7 ± 50.0 ng/μl and 177.2 ± 82.3 ng/μl for Trizol^®^ LS, miRNeasy^®^and miRCURY™, respectively. Curiously, the small RNA fraction, as evaluated by the Qubit^®^ microRNA assay, represented up to 50% of total RNA in the samples. These data are in contradiction with the results obtained with the Agilent Small RNA kit and imply that other majority RNA or DNA species may also be detected by the Qubit^®^ microRNA assay. Since both miRNeasy^®^ and miRCURY™ kits remove most of the DNA present in the samples, a DNA contamination cannot explain the high values observed. On the contrary, despite its selectivity for small RNAs, the Qubit^®^ microRNA assay can also detect 20 to 30% of large RNAs, including ribosomal RNAs (rRNA) and messenger RNAs (mRNA), as described by the manufacturer in its Bioprobes^@^70 journal. Therefore, these data suggest that miRCURY™ kit extracts more large RNA species than miRNeasy^®^ kit. Since the total RNA yield did not differ between both kits (Table 1), our data imply that miRCURY™ kit favours the isolation of the majority rRNAs (and maybe also mRNAs) over miRNAs, thus explaining the high values obtained with the Qubit^®^ assay and the low recovery of miRNAs observed by RT-qPCR and by the Agilent Small RNA kit.

### Performances of RNA extraction methods in the isolation of miRNAs from plasma and bodily fluid-derived exosomes for RT-qPCR analyses

Due to the increased interest in the detection of miRNAs as non-invasive biomarkers in bodily fluids, we then compared the efficacy of miRCURY™ and miRNeasy^®^ kits in the isolation of miRNAs from plasma, plasma exosomes and urinary exosomes.

**Figure 3:**
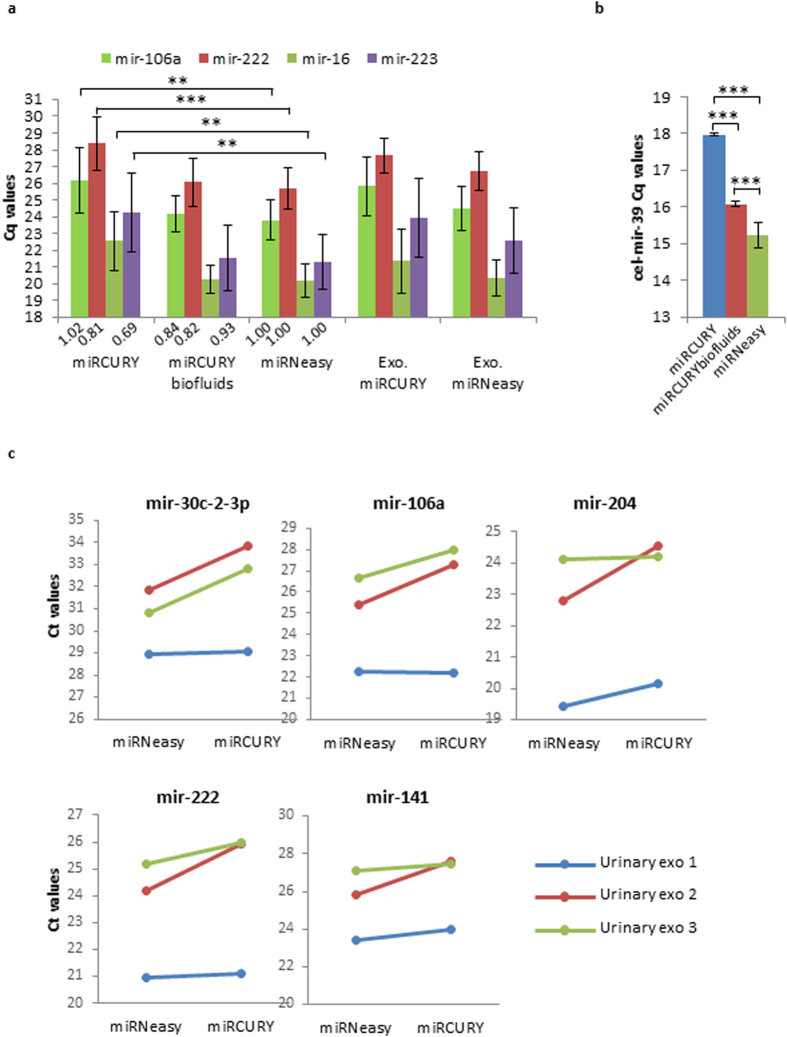
Detection of microRNAs in bodily fluids by RT-qPCR, using different RNA extraction protocols and fixed RNA volumes. (**a**) RNA was extracted from 200 μL of plasma (n = 6) using either miRCURY™, miRCURY™ biofluids or miRNeasy^®^ protocol. Exosomes were isolated from 2 × 2 mL of plasma (n = 3), then subjected to RNA isolation by either miRNeasy^®^ or miRCURY™ kit. The results represent mean Cq values ± SD obtained for mir-106a, mir-222, mir-16 and mir-223, using 2.5 μL of RNA/RT reaction. Expression levels of plasma mir-106a, mir-222 and mir-223 were normalized to mir-16 levels and expressed as fold change relative to miRNeasy^®^ condition under the histograms. (**b**) RNA was isolated from 200 μL of plasma (n = 3) containing 25 fmol of cel-mir-39-3p as a spike-in control directly added into the lysis solution before mixing it with the plasma sample. RNA was isolated with either miRCURY™, miRCURY™ biofluids or miRNeasy^®^ protocols. The results represent mean Cq values ± SD obtained for the exogenous cel-mir-39, using 2.5 μL of RNA/RT reaction. (**c**) 50 to 100 mL of urine were used for urinary exosome isolation in two equal fractions. RNA was then extracted by either miRNeasy^®^ or miRCURY™ kit. The results represent the Cq values obtained for mir-30c-2-3p, mir-106a, mir-204, mir-222 and mir-141, generated from 3 different urinary exosome samples, using 2.5 μL of RNA/RT reaction. **P <* 0.01* ***P <* 0.001.*

The results shown in Fig. 3a indicate that the recovery from 200 μL-plasma of all four miRNAs tested was significantly higher with miRNeasy^®^ versus miRCURY™ (*P < 0.01*). In contrast, the miRCURY™ biofluids and the miRNeasy^®^ kits showed comparable miRNA recovery (Fig. 3a). Similarly, in plasma exosomes, RNA extraction with miRNeasy^®^ resulted in slightly better miRNA detection than miRCURY™ (Fig. 3a). The highly present endogenous mir-16 is commonly used as an internal control to normalize miRNA levels in plasma^1^. Thus we represented, as numbers displayed under the histograms in Fig. 3a, the expression levels of plasma mir-106a, mir-222 and mir-223 when normalized to mir-16 levels and expressed as fold change relative to miRNeasy^®^ condition. As expected, these fold change values are not representative of the real levels of each miRNA since the recovery of the normalizer itself varies with the RNA isolation method.

Exogenous spike-in controls are often used to monitor the efficiency of RNA extraction. Therefore, we assessed the recovery, by each of the RNA isolation methods, of the exogenous control cel-mir-39-3p spiked into 200 μL of plasma. As shown in Fig. 3b, the recovery of cel-mir-39-3p was best achieved with miRNeasy^®^ followed by miRCURY™ biofluids, whereas cel-mir-39-3p was detected at significantly lower levels with miRCURY™ isolation method. In order to investigate if the differences in miRNA recovery of the kits is related to the sample volume, we then assessed miRNA detection from 50 μL, 100 μL and 200 μL of plasma, using each of the RNA extraction methods. As shown in Supplementary Fig. S7, when using 50 μL of plasma, the best miRNA detection was achieved with miRNeasy^®^ isolation, while both Exiqon kits showed lower performance (*P* < *0.001*). With 100 μL of plasma, miRNeasy^®^ and miRCURY™ biofluids kits demonstrated equal efficiencies in miRNA recovery and outperformed miRCURY™ kit (*P* < *0.001*). With 200 μL of plasma, the recovery of miRNAs continued to increase with both miRNeasy^®^ and miRCURY™ biofluids (lower Cq values when compared to 100 μL-plasma). Nevertheless, contrarily to miRCURY™ biofluids, the recovery of miRNAs (with the exception of mir-222) with miRNeasy^®^ tended to reach a plateau, as the recovered amount of miRNAs was not anymore proportional to the input volume of plasma, thus suggesting a slight column saturation. It is important to mention that these results were obtained from 3 plasma samples. In these experiments, miRCURY™ biofluids permitted a slightly better miRNA detection than miRNeasy^®^ from 200 μL of plasma. However, when these data are combined with 3 additional plasma samples to yield the results presented in Fig. 3a, the differences observed between miRCURY™ biofluids and miRNeasy^®^ are not anymore significant. The efficacy of miRCURY™ kit in miRNA recovery was not satisfactory with any of the starting plasma volumes.

The performance of miRNeasy^®^ and miRCURY™ kits was also evaluated in exosomes from urine samples. In order to exclude inter-individual variabilities due to differences in water excretion into urine, we chose to represent individual data from urinary exosomes obtained from 3 healthy donors, rather than the mean ± SD of the data. For each of the 5 miRNAs analyzed, miRNeasy^®^ permitted a better miRNA detection in 2 out of 3 samples, whereas similar recovery by the two kits was observed in the third sample (Fig. 3b).

Altogether these data demonstrate that, when compared to miRCURY™, miRNeasy^®^ allowed a better detection of miRNAs from plasma and from exosomes isolated from plasma and urine. Moreover, both miRNeasy^®^ and miRCURY™ biofluids permitted a satisfactory miRNA detection from plasma.

### Impact of the RNA extraction method on miRNA profiling in plasma and bodily fluid-derived exosomes using Taqman Low Density Arrays (TLDA)

**Figure 4:**
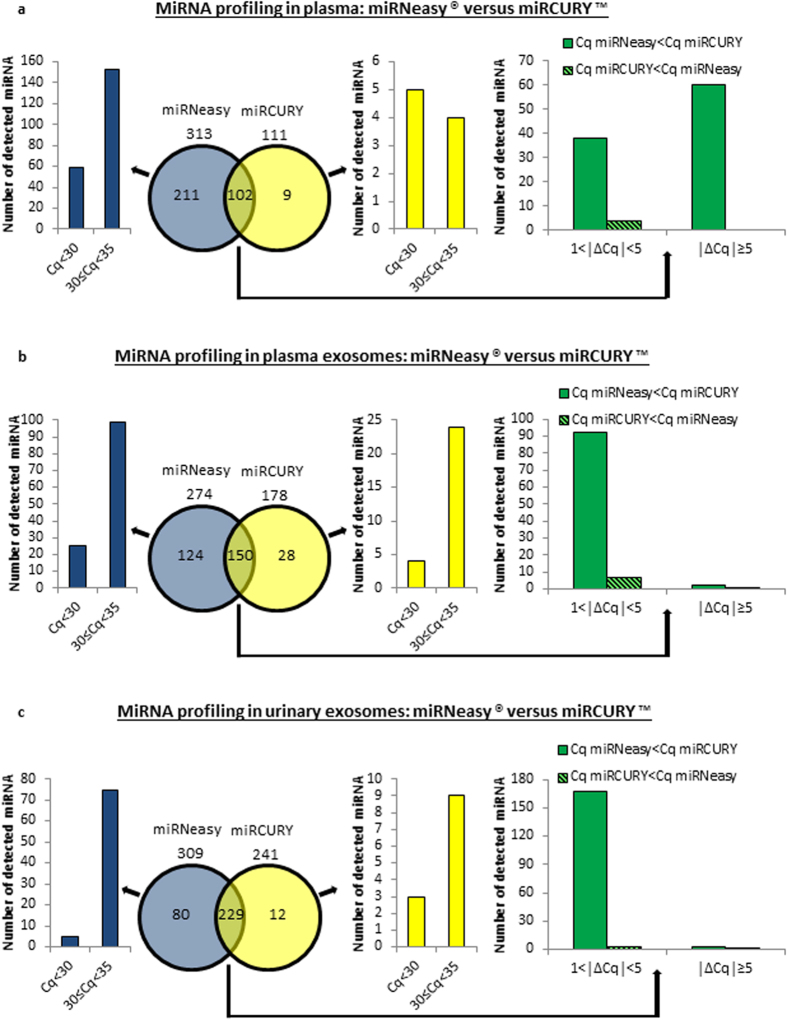
Impact of the RNA extraction method on miRNA profiling in plasma and bodily fluid-derived exosomes, using TLDA. The Venn diagrams compare the numbers of unique and overlapping miRNAs, detected by TLDA, in RNA samples extracted with either miRNeasy^®^ or miRCURY™ kit. The comparison miRNeasy^®^ versus miRCURY™ was performed in (**a**) plasma, (**b**) plasma exosomes and (**c**) urinary exosomes. RNA extraction was done using (**a**) 200 μL of plasma, (**b**) exosomes originating from 2 mL of plasma and (**c**) exosomes isolated from 25 to 50 mL of urine. For each biological source (each panel represents data from one biological source, i.e., from plasma, plasma exosomes or urinary exosomes), two histograms display the number of miRNAs detected at high (Cq < 30) or medium levels (30 ≤ Cq < 35) by either one technique or the other. The third histogram indicates the number of miRNAs detected when using both kits, with Cq differences (**Δ**Cq) between 1 and 5 or superior to 5. These overlapping miRNAs may be detected at higher levels with either miRNeasy^®^ (Cq miRNeasy < Cq miRCURY) or miRCURY™ kit (Cq miRCURY < Cq miRNeasy). The miRNA profiles were generated on TLDA using 3 μl of RNA/RT reaction. One experiment from each of plasma, plasma exosomes and urinary exosome samples is shown.

In order to determine whether the differences observed by RT-qPCR for selected miRNAs are common to other miRNAs when using miRNeasy^®^ and miRCURY™ kits, we broaden these analyses by comparing the miRNA profiles in plasma, plasma exosomes and urinary exosomes using TLDA and each of the extraction methods. Comparisons are made between the miRNeasy^®^ and miRCURY™ extractions in each biological source separately and not between the different sources. Only miRNAs with Cq < 35 were considered as detected. Among the 756 miRNAs covered by the arrays, 313, 274 and 309 miRNAs were detected in RNA samples isolated with miRNeasy^®^ compared with 111, 178 and 241 miRNAs detected using miRCURY™ kit, in plasma, plasma exosomes and urinary exosomes, respectively (Fig. 4). Only a small subset of miRNAs were detected exclusively with miRCURY™ extraction (9, 28 and 12 miRNAs in the 3 samples analyzed). On the contrary, a considerable amount of miRNAs were specifically detected with miRNeasy^®^ (211, 124 and 80 miRNAs in the samples analyzed). Importantly, in the set of common miRNAs, the expression levels of miRNAs isolated with miRNeasy^®^ were in general higher than those observed with miRCURY™, with Cq differences exceeding 5 in some cases (Fig. 4). Moreover, among the miRNAs detected only after extraction by one technique or the other, some miRNAs were expressed at relatively high levels (Cq < 30), suggesting that the isolation of at least some miRNAs is closely dependent on the extraction method.

Overall, these results show that a larger number and amount of miRNAs can be isolated with miRNeasy^®^ when compared to miRCURY™, but that some selected miRNAs can be preferentially or exclusively extracted by one method or the other.

## Discussion

In the last decade, the research towards the discovery of miRNA-based biomarkers has generated a great interest in the medical and scientific communities^5^,^7^,^20^. Nevertheless, lots of technical issues should be considered before the translation of miRNAs into the daily clinical practice. To this regard, several groups have performed comparative studies^11^,^12^,^14^,^21^,^22^ in order to determine the most suitable RNA extraction protocols for miRNA analysis. Still, no consensus has been found as illustrated by Supplementary Table S1 that recapitulates the widespread conclusions obtained from main comparative studies.

In this article, we have shown that miRNA recovery from different sample types is largely influenced by the RNA isolation method used. Whereas miRCURY™ kit permitted the isolation of highly pure RNA, of better quality when compared to miRNeasy^®^ and Trizol^®^ LS, resulting in an optimal RT-qPCR efficiency, the recovery of miRNAs by this method was dramatically affected by the amount of starting material. In fact, our results suggest that miRCURY™ columns tend to be saturated by large RNA species when the starting number of cells increases, thus impeding the optimal recovery of miRNAs. This conclusion is further corroborated by the decreased miRNA recovery following the addition of the MS2 RNA as a carrier when using miRCURY™ kit and a low cell number. Moreover, the same quantity of miRCURY™-isolated RNA displayed better results as for miRNA detection, when the starting cell number was 200 × 10^3^ rather than 800 × 10^3^ (Fig. 2), supporting the column saturation hypothesis. Interestingly, despite the low purity of Trizol^®^ LS-extracted RNA, this method outperformed miRCURY™ kit when using high input material as demonstrated by the RT-qPCR results. Importantly, when using as few as 100 or 1000 cells, miRNeasy^®^ kit permitted a better miRNA detection when compared to miRCURY™ kit. This observation cannot be attributed to a column clogging and suggests a less efficient adsorption capacity of miRCURY™ column when the amount of input material is very low. With 10 × 10^3^ and 25 × 10^3^ cells, both kits performed equally regarding miRNA recovery, most likely because the amount of input material is no longer limiting while staying below the critical saturation point. It is important to mention that the reduced recovery of miRNAs by the miRCURY™ kit should not be considered as a reduced ability of the kit for the isolation of all small RNA species, since the detection of U6 snRNA from miRCURY™-isolated RNA proved akin to or slightly better than its detection from samples isolated by the other methods investigated. These findings suggest that miRNAs might be the most affected by the sub-optimal capacity of the columns.

The superior performance of miRNeasy^®^ kit, in the limits of the amounts of input material we have used, resulted in a higher miRNA yield not only from cells *in vitro* but also from increasing volumes of plasma and from biofluid-derived exosomes. Nevertheless, our data demonstrate a comparable ability of miRNeasy^®^ and miRCURY™ biofluids to recover miRNAs from 100 μL and 200 μL of plasma, whereas the detection of miRNAs from 50 μL was better achieved with miRNeasy^®^. Interestingly, McAlexander M *et al.* showed that increasing the starting plasma volume led to a less efficient recovery of miRNAs by miRCURY™ biofluids kit, due to protein-mediated clogging of the columns and/or increased quantity of blood-borne inhibitors of RT-PCR^21^. In our hands, such decrease in miRNA recovery when doubling the plasma volume was not observed with miRCURY™ biofluids by rather with miRNeasy^®^. This discrepancy may be due to the density and the intrinsic composition of each plasma which may vary from one sample to another, depending, for example, on the collection time (pre- or post-prandial), and thus may differently affect the adsorption of miRNAs to the columns of each kit. Accordingly, we noticed that the best miRNA recovery from 200 μL-plasma was observed with either miRNeasy kit or miRCURY™ biofluids kit. The low performance of miRCURY™ kit in miRNA recovery from 50 μL to 200 μL of plasma suggests poor column adsorption ability, carryover of blood-borne RT-PCR inhibitors, and/or column clogging. Besides, in accordance with previous findings^12^, our data on exosomal miRNAs do not support the hypothesis proposed by others claiming that column-based methods, such as miRCURY™ kit, may be more suitable than the phenol (including combined phenol and column)-based techniques for extracting exosome-derived RNA because of the particular lipid composition of exosomal membranes^8^,^23^. In this regard, Moldovan L *et al.* described an optimized protocol for the analysis of miRNA from plasma- and serum-derived extracellular vesicles/exosomes using the miRNeasy^®^ kit^24^. Exosomes carry specific sets of lipids, proteins and RNAs^25^,^26^. The exosomal RNA repertoire contains miRNAs (10 to 40%) and other small non-coding RNAs, natural antisense RNAs, tRNAs, mRNAs, rRNAs and long non-coding RNAs^12^,^27^,^28^. The extent to which each exosomal component participates to the differential recovery of miRNA by the miRNeasy^®^ and the miRCURY™ kits is still to be investigated.

Our results demonstrated that higher amounts of miRNAs can be detected with miRNeasy^®^ compared to miRCURY™, using single RT-qPCR miRNA assays. Whereas miRNA profiling using TLDA confirmed the overall superiority of miRNeasy^®^ in isolating miRNA from plasma, plasma exosomes and urinary exosomes, it also showed that some selected miRNAs can be preferentially or exclusively extracted by either of the methods. Differences in miRNA profiling have been previously observed when comparing the NucleoSpin^®^ miRNA plasma and the miRNeasy^®^ kits in TLDA assays^16^, and when comparing Trizol^®^, mirVana™ (Life technologies) and miRNeasy^®^ kits in miRNA microarrays^29^. Differences in GC content of miRNAs^30^ and the presence of variable amounts of proteins and lipids in the lysates^16^ have been proposed to affect the nature of the extracted miRNAs.

In the study of Eldh M *et al.*, Trizol^®^ and miRNeasy^®^ were less efficient than miRCURY™ for total RNA isolation from cells, whereas all three methods extracted small RNAs, including miRNAs, with equal ability^23^. These data are in contrast with our findings demonstrating that miRCURY™ and miRNeasy^®^ are equally efficient in total RNA isolation and are superior to Trizol^®^ LS, whereas miRNeasy^®^ resulted in the highest small RNA, including miRNA, yield. These discrepancies may generate, at least in part, from the different amounts of cells used in both studies. Moreover, with the miRNeasy^®^ kit, we opted to use Phase Lock Gel (PLG) for the collection of the aqueous phase in order to increase the volume retrieved while getting a better RNA purity. This may also explain the higher RNA yield we have obtained compared to the study of Eldh M *et al.* Interestingly Trizol^®^ LS permitted the highest enrichment of miRNA in the small RNA fraction when the extractions were done with a small cell number (miRNA/small RNA = 18.5%, see Table 4). Nevertheless, the low RNA purity obtained with Trizol^®^ LS did not yield satisfactory RT-qPCR results. Our findings are in accordance with previous studies demonstrating the superiority of miRNeasy^®^ kit over Trizol^®^ in miRNA detection^11^,^31^. However, another study reported that Trizol^®^ LS proved more efficient than mirVana™PARIS™ (a combined phenol and column-based technique) in the extraction of miRNA from serum^14^. These findings may sound contradictory; however, we have demonstrated that the amount of input material can play a major role in such disparate results. Indeed our results obtained with high starting cell number showed a better detection of miRNA with Trizol^®^ LS versus miRCURY™ extraction, in contrast to the results obtained with a low cell number.

In conclusion, we have shown that the column-based method of the miRCURY™ kit has the advantage of providing highly pure RNA; However its sub-optimal column capacity has the inconveniency of poor miRNA recovery when using very low amounts of cells and plasma and rapid saturation by large RNA species and plasma components, thus hindering the isolation of miRNAs. On the contrary, the combined phenol and column-based method of the miRNeasy^®^ kit displayed a better performance as for miRNA detection by RT-qPCR, despite a less optimal RNA purity. However, one should keep in mind that this conclusion is peculiar to miRNA isolation and should not be considered as a general statement relative to any other RNA species. The miRCURY biofluids kit demonstrated satisfactory results in miRNA isolation from 100 μL and 200 μL of plasma. Concerning the phenol-based method of Trizol^®^ LS, it resulted in RNA extracts with very low purity which affected RT-qPCR efficiency, albeit acceptable results were obtained with high input material. Nevertheless, we do not recommend this method for miRNA studies owing to (1) the reproducibility issues that may arise from the poor sample purity, (2) the previously reported selective loss of small RNA molecules with low GC content^30^, and (3) the availability of other methods demonstrating better results for miRNA analysis.

Finally, when performing miRNA analysis, one should always consider the issues that may arise from the use of random amounts of starting material. Hence it is highly advisable to carefully choose the RNA isolation method according to the type and quantity of input material, and to the downstream experimental requirements. In this regard, we showed that miRNeasy^®^ kit is efficient in extracting miRNAs from cells in culture, plasma and biofluid-derived exosomes. However, due to the simplicity of its execution, rapidity and phenol-free procedure, one may prefer to use miRCURY™ biofluids for the isolation of miRNA from 100 μL and 200 μL of plasma. Last but not least, it is of major importance to strictly adhere to the same RNA technique AND to the same amount of input material throughout the entire study in order to have comparable measurements.

## Methods

### Cell culture conditions, study population and sample collection

A549 cells were cultured in DMEM medium (Lonza, Verviers, Belgium), containing 10% fetal bovine serum and were incubated at 37 °C in an atmosphere of 5% CO_2_.

The subjects were healthy donors who signed an informed consent according to the Declaration of Helsinki. All the experimental protocols were approved by the national research ethics committee in Luxembourg “Comité National d’Ethique de Recherche” (CNER), and the study was authorized by the national commission for data protection “Commission Nationale pour la Protection des Données” (CNPD). Peripheral blood samples (n = 6) were drawn into EDTA-containing tubes and centrifuged at 1000 g for 10 min at 4 °C. The supernatants were then centrifuged at 2000 g for 10 min at 4 °C to remove debris. The plasma supernatants were aliquoted in 2-mL tubes and stored at -80 °C.

Midstream spot urine (n = 3) (50 to 100 mL) was collected in sterile containers supplemented with a protease inhibitor cocktail (Complete Protease Inhibitor, Roche Diagnostics, Vilvoorde, Belgium) and immediately processed for exosome isolation.

### Isolation of plasma exosomes

Four mL of plasma were diluted 5 times with phosphate buffered saline (PBS), then centrifuged at 10,000 g for 50 min at 4 °C to remove large extracellular vesicles. The supernatant was sequentially passed through 1.6- and 0.2-μm pore-sized filters to remove remaining debris and large particles. The diluted plasma was equally split in two tubes and ultracentrifuged at 110,000 g for 1 h 30 min at 4 °C to collect exosomes. Exosomes were then washed with PBS, pelleted again at 110,000 g for 1 h 30 min at 4 °C and resuspended in PBS.

### Isolation of urinary exosomes

Urinary exosomes were isolated as previously described^32^,^33^. Briefly, urine was sequentially centrifuged at 1000 g for 15 min at 4 °C and at 17,000 g for 30 min at 4 °C to remove cells, cellular debris and large particles. The resulting supernatant containing exosomes and microvesicles (MV) was reserved as S1. The 17,000 g pellet was resuspended in Tris 20 mM pH 8.6 to release exosomes entrapped in the Tamm-Horsfall protein polymer and centrifuged again at 17,000 g for 30 min at 4 °C. The resulting supernatant (S2) was added to S1, split in two equal fractions and ultracentrifuged at 200,000 g for 1 h 10 min to pellet exosomes/MV (hereafter referred to as exosomes). Exosomes were washed with Tris 20 mM pH 8.6 and subjected to RNA extraction.

### RNA extraction

Total RNA was isolated from 100, 1000, 10 × 10^3^, 25 × 10^3^ (25c), 200 × 10^3^ (200c) and 800 × 10^3^ (800c) cells (the last three conditions were sometimes referred to as low, medium and high cell numbers/densities, respectively), from 50 μl, 100 μl and 200 μl of plasma, and from biofluid-derived exosomes, using Trizol^®^ LS reagent (Life Technologies, Gent, Belgium), miRNeasy^®^ Mini kit (Qiagen, Venlo, Netherlands), miRCURY™ RNA Isolation and miRCURY™ biofluids kits (Exiqon, Vedbaek, Denmark) as indicated in the manuscript. RNA isolation was performed as recommended by the manufacturers. MS2 RNA (Roche Diagnostics, Vilvoorde, Belgium) was added as a carrier for RNA extraction from plasma, biofluid-derived exosomes, and, when indicated, from A549 cells. When specified, 25 fmol of the spike-in control *Caenorhabditis elegans* cel-mir-39-3p (mirVana™ miRNA mimic, Life Technologies, Gent, Belgium) were added per extraction into the lysis solution of each method prior to the combination with 200 μl of plasma. MS2 was mixed with the QIAzol (1000 μl/sample for plasma and plasma exosomes, and 700 μl/sample for A549 cells and urinary exosomes) and with Exiqon kits lysis solutions prior to the addition of the samples. The quantities of MS2 used were 0.64 μg/sample of A549 cells and urinary exosomes, and 0.96 μg/sample of plasma and plasma exosomes. A “no cell” condition containing 0.64 μg of MS2 and subjected to the same miRNeasy^®^ protocol used for A549 cells has been included. Phase Lock Gel™ (PLG) tubes (5 Prime, Hilden, Germany) were used with the miRNeasy^®^protocol for the recovery of the aqueous phase. RNA was eluted in 50 μL of RNase-free water and stored at −80 °C.

### Assessment of RNA quantity and quality

Total RNA was quantified using NanoDrop ND-1000 spectrophotometer (Isogen Life Science, Temse, Belgium). RNA purity was assessed by checking the absorbance at 260 nm, 280 nm and 230 nm. An A260/A280 ratio <1.8 denotes protein contamination and an A260/A230 <2 indicates presence of phenol, chaotropic salts (guanidinium thiocyanate) or proteins. RNA integrity was analyzed with the Agilent RNA 6000 Nano or Pico kits (using 1 μL of RNA from the stock solution or from equally diluted samples) on the Agilent Bioanalyzer 2100 (Agilent technologies, Diegem, Belgium) and evaluated based on the RNA Integrity Number (RIN) that classifies the RNA as very degraded (RIN = 1) to intact (RIN = 10). The quantity and the profile of small RNAs were assessed by capillary electrophoresis and fluorescence, using the Agilent Small RNA kit on the Agilent Bioanalyzer 2100, and the Qubit^®^ microRNA assay (Life Technologies, Gent, Belgium) on the Qubit^®^ 2.0 Fluorometer. For Agilent Small RNA assay, 1 μL of RNA from the stock solution or from equally diluted samples was used. For Qubit^®^ microRNA assay, 10 μL of equally diluted RNA samples were used. Electropherograms were visualized using the Agilent 2100 Expert software (Agilent technologies).

### Analysis of miRNA gene expression using TaqMan^®^ MicroRNA Assays

The detection of miRNAs was performed by RT-qPCR using TaqMan^®^ MicroRNA Assays. Briefly, 2 μL of diluted (or 2.5 μL of undiluted) RNA solution (see below for further details) or 5 ng of RNA were used in a 7.5 μL-RT reaction. Real-time PCR reactions were performed with 0.67 μL of the RT product in 10 μL-PCR reaction volume and done in triplicate using the ViiA^TM^ 7 Real-Time PCR System (Life Technologies, Gent, Belgium). RT and PCR reactions were performed following the programs recommended by the TaqMan^®^ MicroRNA assays.

### Quantities of input material used for RT reactions, and considerations for the interpretation of PCR results from A549 cells

For RT-qPCR experiments using RNA from plasma, plasma exosomes and urinary exosomes, 2.5 μL of RNA samples were used in the RT reactions.

For RT-qPCR experiments using RNA from A549 cells: When a constant amount of RNA was used, 5 ng were included in the RT reaction. When a fixed volume of RNA was used, 2 μL of diluted or 2.5 μL of undiluted RNA solution were used according to the following considerations:

When using 100, 1000 and 10 × 10^3^ cells as input amounts, 2.5 μL of undiluted RNA solution were used in the RT reactions.

When using 25 × 10^3^, 200 × 10^3^ and 800 × 10^3^ cells as input amounts, for mir-106a, mir-222 and U6 snRNA analyses, in order to decrease the influence of PCR inhibitory factors (such as phenol) that may be found in some RNA samples, and to follow the instructions of the RT kit as for the RNA quantity to use, we first diluted the RNA samples as follows :

Since the recommended input RNA quantity to be used with the TaqMan^®^ MicroRNA RT kit is 0.5 to 5 ng of RNA/7.5 μl of RT reaction, the RNA samples were first diluted (the same dilution factor was applied to all the samples of the same experiment containing the same starting cell number) to a maximal concentration of 2.5 ng/μl; 2 μl of the diluted samples were used in the 7.5 μl-RT reaction volume. The mean dilution factors were then 21.7, 66.7 and 150 for the 25c, 200c and 800c series, respectively. When taking into account that the 800c series contains 4 times more cells than the 200c series which contains 8 times more cells than the 25c series, the RT-qPCR results obtained with a fixed RNA volume reflect data from: 800c samples 1.78 times more concentrated than the 200c samples, and 200c samples 2.6 times more concentrated than the 25c samples. Assuming a PCR amplification efficiency of 100%, 1.78 and 2.6 fold differences in starting template concentration should give cycle threshold (Cq) differences of 0.83 between the 800c and the 200c samples (2^0.83^ = 1.78), and 1.38 between the 200c and the 25c samples (2^1.38^ = 2.6).

Since mir-141 is expressed at very low levels in A549 cells, the 2 μl of diluted RNA resulted in undetermined Cq; hence we used 2.5 μl of undiluted RNA from the 25c, 200c and 800c samples as starting material for mir-141 detection. The PCR reactions performed with the 25c samples generated Cq > 35 and thus were not considered. As the 2.5 μl of undiluted 800c RNA samples contain 4 times more starting material than the 2.5 μl of undiluted 200c RNA samples, the theoretical mir-141 Cq difference should be of 2 (2^2^ = 4) between these too conditions.

The data were analyzed by the ΔΔCq method. The fold change (2^−**ΔΔ**Cq^) is the normalized gene expression (2^−**Δ**Cq^) in the sample divided by the normalized gene expression (2^−**Δ**Cq^) in the control, as indicated in the manuscript.

### miRNA profiling using TaqMan Low-Density Arrays (TLDA)

A broad miRNA profiling of plasma and biofluid-derived exosomes covering 756 miRNAs was carried out using TaqMan^®^ MicroRNA Arrays A and B (Life Technologies, Gent, Belgium) as described by the manufacturer. Briefly, 3 μL of RNA extracts were reverse-transcribed using Megaplex RT primer pools A and B in a final volume of 7.5 μL. cDNA targets were then preamplified using 2.5 μL of the RT product in a 25 μL-preamplification reaction. Nine μL of 4 time-diluted preamplification products were included into the 900 μL-PCR reaction required for each TaqMan^®^ MicroRNA Array. Real-time PCR was performed using a ViiA^TM^ 7 Real-Time PCR System (Life Technologies, Gent, Belgium). Only miRNAs with Cq < 35 were considered as detected.

### Statistical analysis

Statistical analysis was carried out with the R software, using a two-way ANOVA model with experiment and extraction method as factors, followed by Fisher’s least significant difference (LSD) test. The p values of the fold change were calculated based on a Student’s t-test of the replicate 2^−**Δ**Cq^ values. A *P*-value < 0.05 was considered statistically^31^,^32^,^33^ significant.

## Additional Information

**How to cite this article**: El-Khoury, V. *et al.* Assessing cellular and circulating miRNA recovery: the impact of the RNA isolation method and the quantity of input material. *Sci. Rep.* **6**, 19529; doi: 10.1038/srep19529 (2016).

## Supplementary Material

Supplementary Information

## Figures and Tables

**Table 1:** Comparison of RNA concentration and integrity using different RNA extraction methods and cell densities.

Method	RNA concentration (ng/μL)			RIN value		
	25,000 cells	200,000 cells	800,000 cells	25,000 cells	200,000 cells	800,000 cells
Trizol LS	29.9 (11.0)	90.2 (27.2)	244.5 (90.4)	9.6 (0.2)	9.5 (0.5)	9.8 (0.1)
miRNeasy	15.1 (3.2)	91.2 (39.1)	339.5 (153.4)	9.6 (0.3)	10.0 (0.0)	9.9 (0.1)
miRNeasy + MS2	35.9 (10.0)	101.5 (46.5)	339.9 (164.2)	6.5 (1.0)	9.1 (0.8)	9.7 (0.3)
miRCURY	15.3 (5.1)	96.9 (48.6)	341.2 (184.4)	9.6 (0.2)	10.0 (0.0)	10.0 (0.0)
miRCURY + MS2	26.3 (5.4)	105.0 (45.1)	346.9 (177.2)	6.5 (0.7)	9.4 (0.6)	9.8 (0.2)

Mean values are shown with standard deviation reported in parenthesis (n = 4).

**Table 2:** Comparison of RNA purity using different RNA extraction methods and cell densities.

Method	Ratio OD (260 nm/280 nm)			Ratio OD (260 nm/230 nm)		
	25,000 cells	200,000 cells	800,000 cells	25,000 cells	200,000 cells	800,000 cells
Trizol LS	1.59 (0.06)	1.76 (0.10)	1.87 (0.06)	0.16 (0.13)	0.33 (0.18)	0.55 (0.15)
miRNeasy	1.70 (0.18)	1.96 (0.07)	2.04 (0.02)	0.16 (0.02)	1.21 (0.26)	1.43 (0.32)
miRNeasy + MS2	1.84 (0.06)	2.01 (0.04)	2.05 (0.02)	0.38 (0.41)	0.90 (0.37)	1.00 (0.37)
miRCURY	1.65 (0.22)	2.01 (0.05)	2.07 (0.02)	0.78 (0.22)	1.66 (0.27)	2.00 (0.18)
miRCURY + MS2	1.83 (0.14)	2.04 (0.03)	2.07 (0.02)	0.97 (0.66)	1.60 (0.54)	1.95 (0.25)

Mean values are shown with standard deviation reported in parenthesis (n = 4).

**Table 3:**
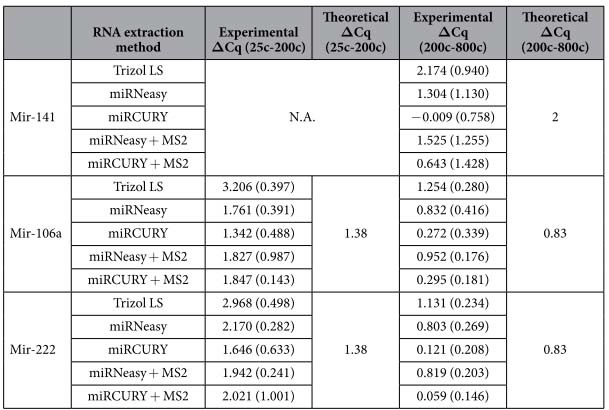
Comparison of experimental and theoretical Cq differences (**Δ**Cq) for the evaluation of miRNA recovery from increasing number of cells using different RNA extraction methods.

Mean values are shown with standard deviation reported in parenthesis (n = 3).

N.A. = not applicable.

**Table 4:** Comparison of small and miRNA recovery using different RNA extraction methods and cell densities.

Cell number	Method	small RNA conc. (ng/μL)	miRNA conc. (ng/μL)	miRNA/small RNA (%)
25,000 cells	Trizol LS	0,81 (0.34)	0,15 (0.06)	18,5 (3.4)
	miRNeasy	1,25 (0.47)	0,17 (0.03)	14,3 (4.1)
	miRCURY	1,15 (0.59)	0,11 (0.07)	9,5 (2.4)
800,000 cells	Trizol LS	20,11 (9.53)	0,64 (0.38)	3,5 (1.3)
	miRNeasy	29,62 (11.88)	1,17 (0.40)	4,3 (0.5)
	miRCURY	17,89 (10.71)	0,62 (0.26)	4,0 (1.8)

Mean values are shown with standard deviation reported in parenthesis (n = 4).
