# Supplementary material for: Assessing cellular and circulating miRNA recovery: the impact of the RNA isolation method and the quantity of input material
Source: Sci Rep. 2016 Jan 20;6:19529. doi: 10.1038/srep19529 (PMC4726450; doi:10.1038/srep19529)
Supplement: Supplementary Information [file srep19529-s1.pdf]

Assessing cellular and circulating miRNA recovery: the impact of the RNA isolation method and the quantity of input material

Victoria El-Khoury, Sandrine Pierson, Tony Kaoma, François Bernardin, and Guy Berchem

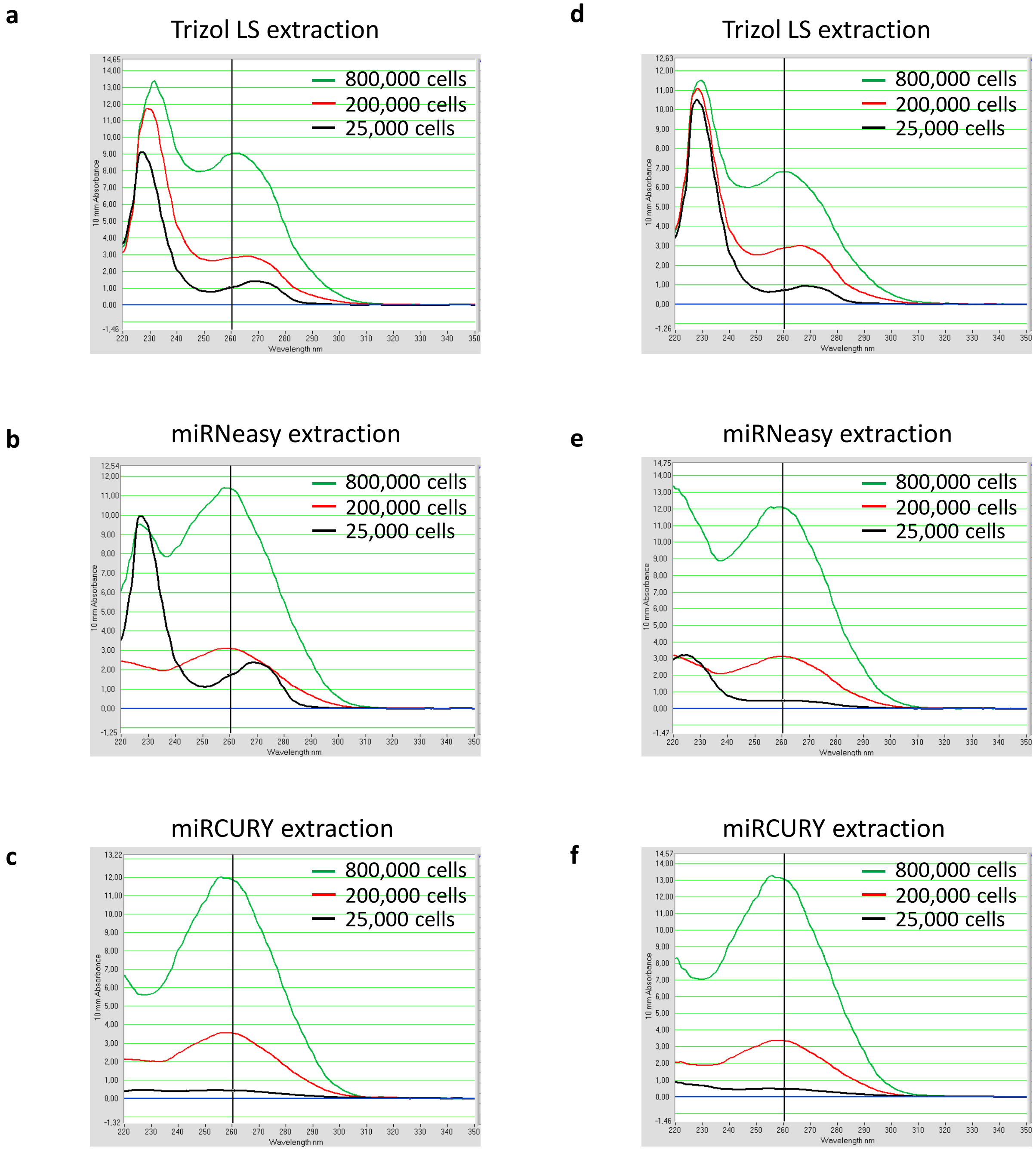

Supplementary Figure S1. Comparison of RNA absorbance spectra obtained from NanoDrop 1000 Data Viewer, corresponding to RNA isolated by either Trizol® LS, miRNeasy® or miRCURY™, from low (25,000 cells), medium (200,000 cells) and high (800,000 cells) cell density samples. Representative data from two independent experiments are shown (a, b and c correspond to one experiment and d, e, and f correspond to another experiment).

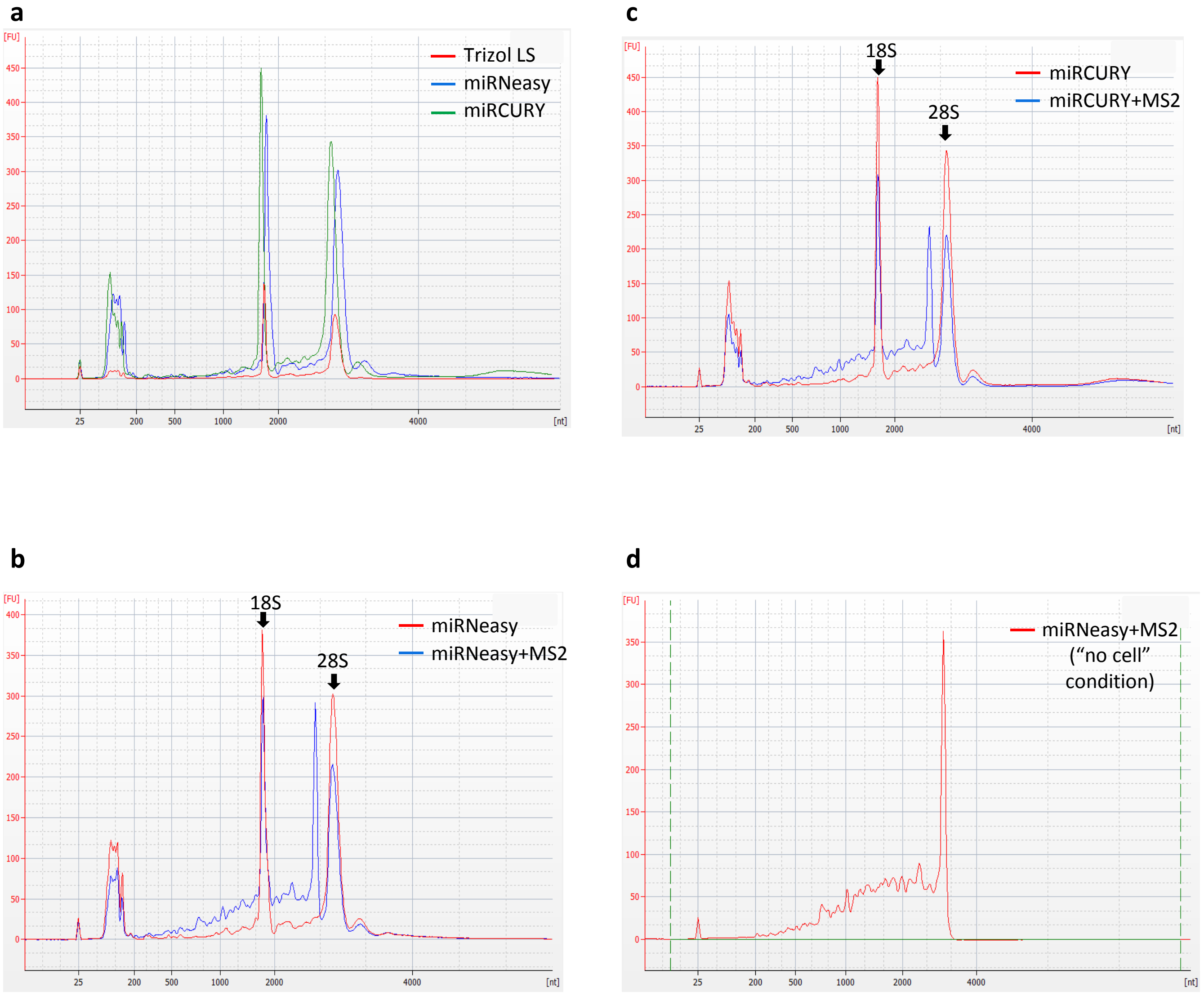

Supplementary Figure S2. Comparison of RNA electropherograms obtained from A549 RNA samples extracted with either Trizol® LS, miRNeasy®, or miRCURY™. RNA from 25 x 10^3 A549 cells was isolated by either of the methods. Electropherograms were obtained from RNA 6000 Pico assay using 1 µL of eluted RNA. A “no cell” condition containing only MS2 RNA was subjected to miRNeasy® extraction. One representative experiment is shown. The electropherogram overlay shows a) the poor extraction efficiency of Trizol® LS over miRNeasy® and miRCURY™ and b and c) the RNA profiles in the presence or absence of the MS2 carrier (0.64 µg/sample) when samples are extracted with miRNeasy® (b) or miRCURY™ (c). The electropherogram of the RNA MS2 carrier without cellular RNA is shown in d.

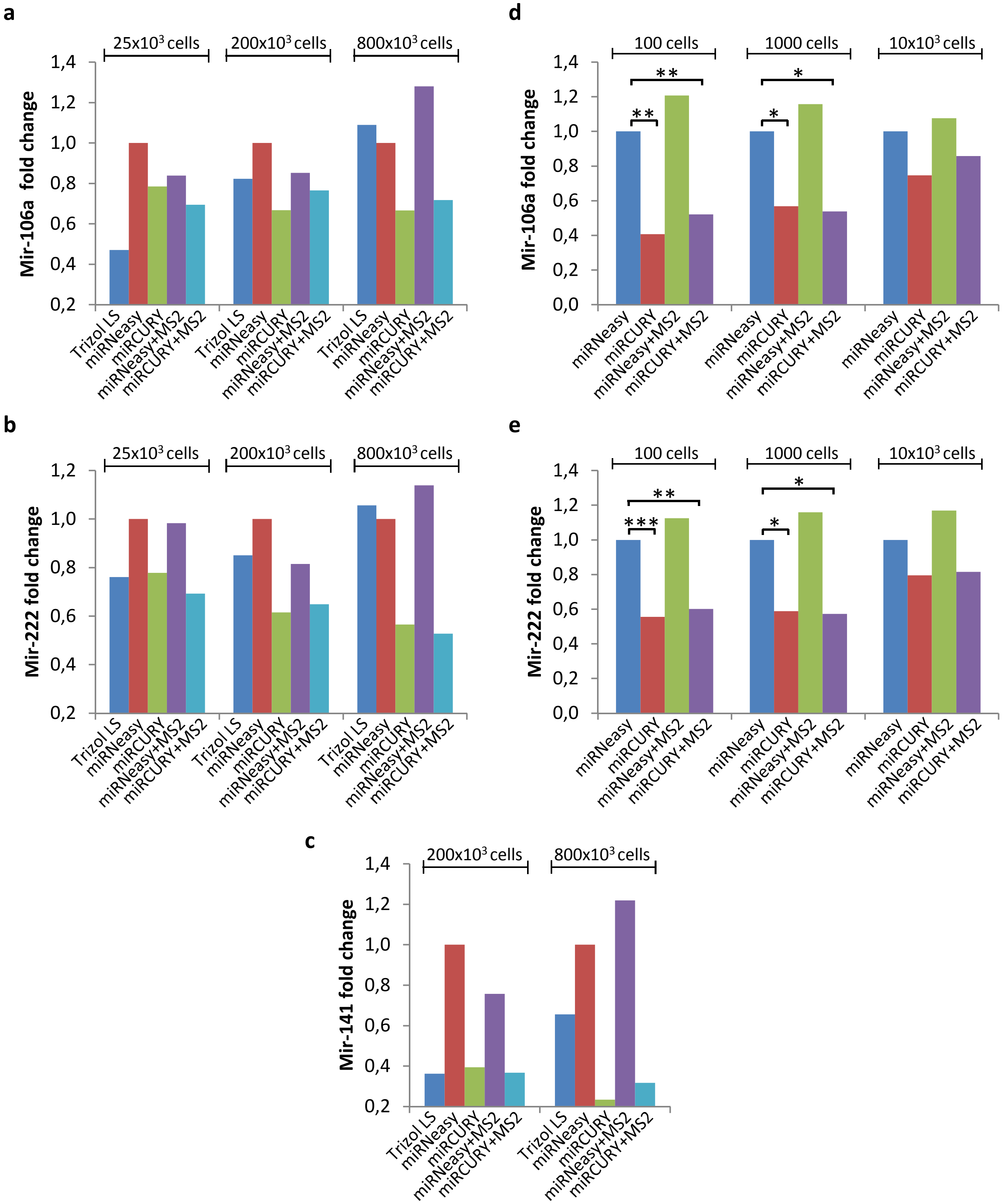

Supplementary Figure S3. MiRNA fold change values using different RNA extraction methods and cell densities, and fixed RNA volumes. Expression levels of (a and d) mir-106a, (b and e) mir-222 and (c) mir-141 were normalized to U6 snRNA levels and expressed as fold change relative to miRNeasy® condition. The detection of miRNAs was performed by RT-qPCR using a fixed volume of RNA samples (see Methods for details). The average fold change of 3 independent experiments is shown. **P < 0.05* ***P < 0.01* ****P < 0.001.*

a

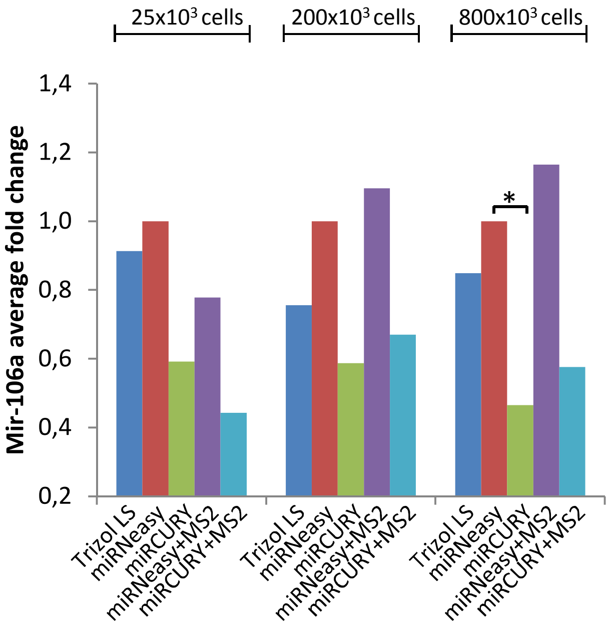

b

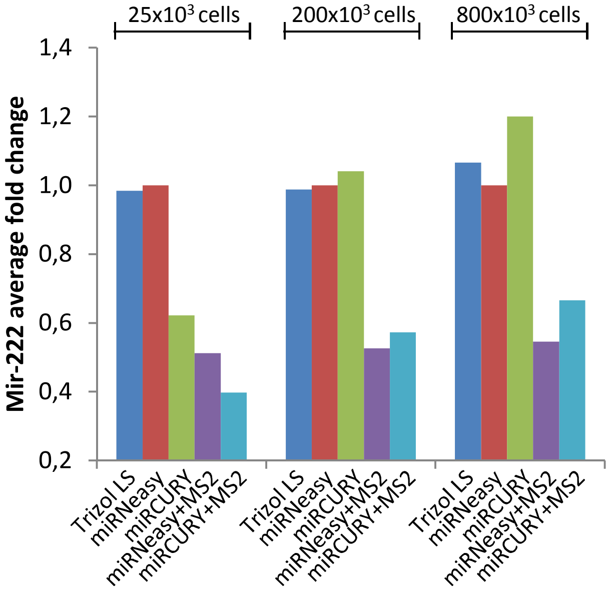

Supplementary Figure S4. MiRNA fold change values using different RNA extraction methods and cell densities, and a fixed RNA quantity. Expression levels of (a) mir-106a and (b) mir-222 were normalized to U6 snRNA levels and expressed as fold change relative to miRNeasy® condition. The detection of miRNA was performed by RT-qPCR using 5 ng of total RNA/RT reaction. The average fold change of 3 independent experiments is shown. *$P < 0.05$

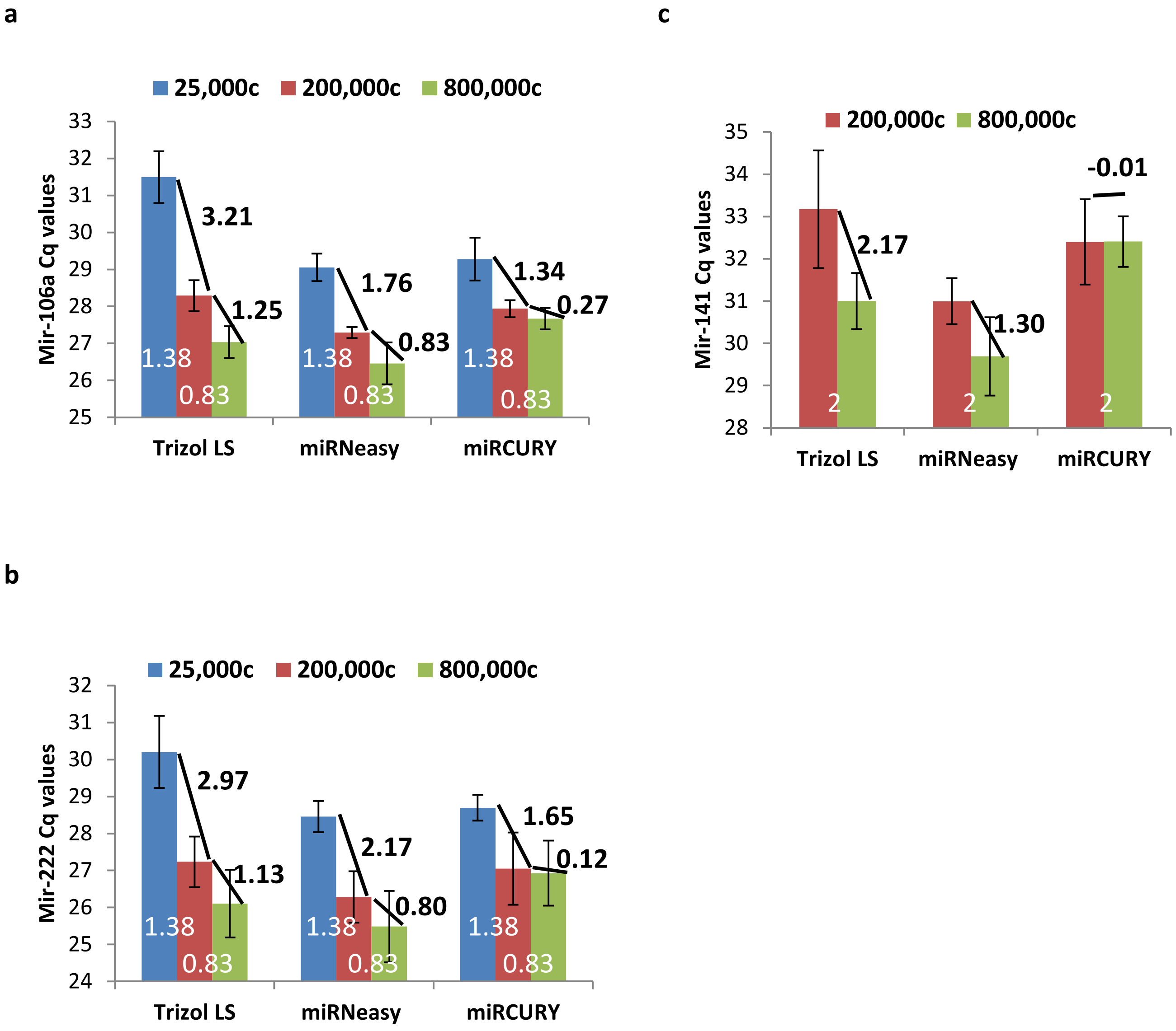

Supplementary Figure S5. Quantitative recovery of miRNA from increasing number of cells using different RNA extraction methods. The histograms represent the recovery by RT-qPCR of a) mir-106a, b) mir-222 and c) mir-141 from 25 x 10^3, 200 x 10^3 and 800 x 10^3 A549 cells subjected to RNA isolation by either Trizol® LS, miRNeasy®, or miRCURY™. A fixed volume of RNA was used for RT reaction. The theoritical Cq differences (ΔCq) between the indicated input amounts are mentioned on the histogram bars in white font, and the experimental Cq differences are shown in black font next to the black line representing ΔCq (see Methods and Table 3 for details). The mean values ± SD of 3 independent experiments are shown.

a

Starting cell number = 25×10^3

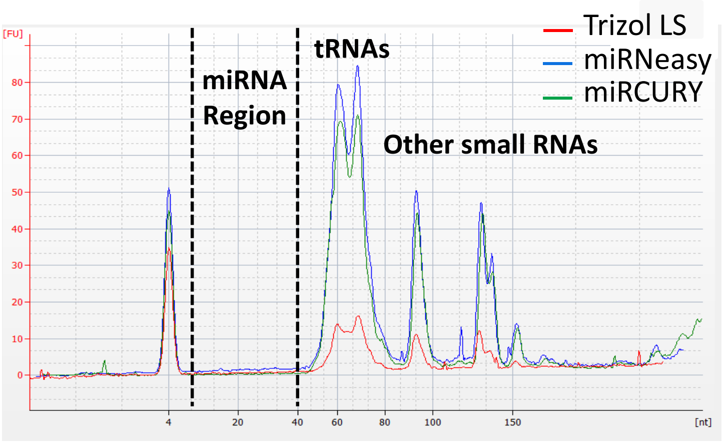

b

Starting cell number = 800×10^3

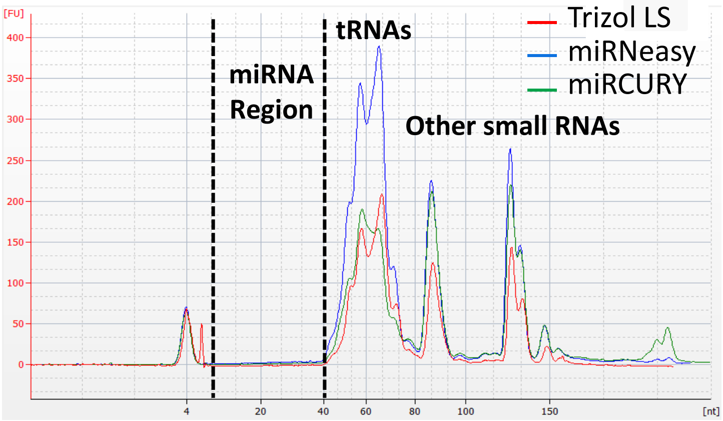

Supplementary Figure S6. RNA electropherogram overlays showing the small RNA fractions obtained with Trizol® LS, miRNeasy®, and miRCURY™ kits, when using different input cell numbers. RNA from a) 25 x 10^3 and b) 800 x 10^3 A549 cells was isolated by either of Trizol® LS, miRNeasy®, or miRCURY™ methods. Electropherograms were obtained using the Agilent Small RNA assay and represent the recovery of the small RNA species including miRNAs with each of the RNA isolation methods. One μL of RNA from the eluted stock solution (a) or from equally diluted samples (b) was used for this assay. One representative experiment is shown.

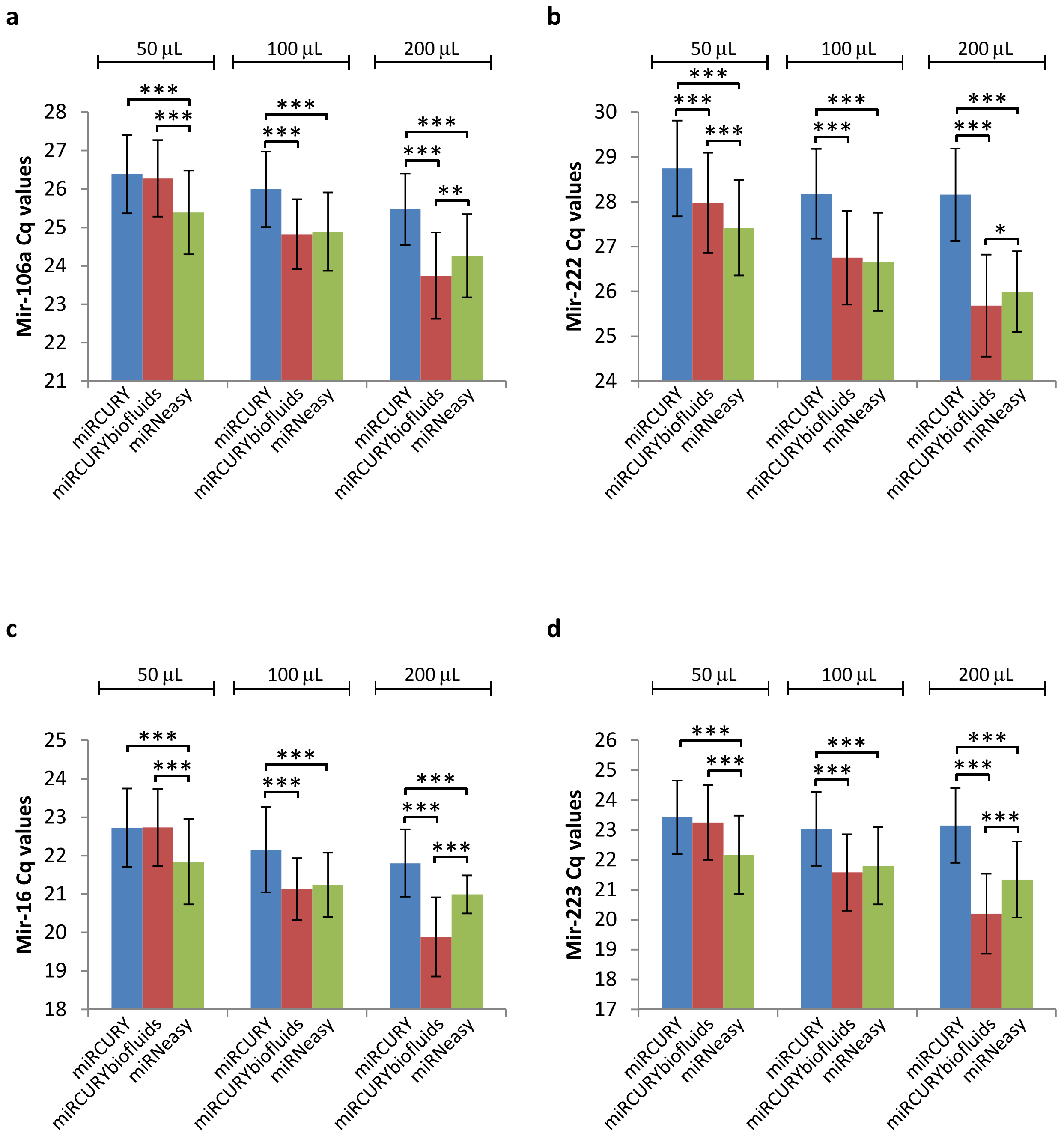

Supplementary Figure S7. Recovery of miRNAs from increasing volumes of plasma using different RNA extraction methods. RNA was extracted from 50 µL, 100 µL and 200 µL of plasma using either miRCURY™, miRCURY™ biofluids or miRNeasy® protocol. The results represent mean Cq values ± SD (n = 3) obtained for (a) mir-106a, (b) mir-222, (c) mir-16, (d) mir-223, using 2.5 µL of RNA/RT reaction. *$P < 0.05$ **$P < 0.01$ ***$P < 0.001$.

Supplementary Table S1. Comparative evaluation of RNA extraction methods in terms of miRNA yield/detection in different sample types across studies.

RNA extraction method																	Sample type	Reference
1	2	3	4	5	6	7	8	9	10	11	12	13	14	15	16	17		
+++		+++				+	++	++++									serum	14
+++		++						+				+					plasma	11
	+		+++	++													plasma	22
	++								+						+++	++++	plasma	21
			+													++	plasma	21
		+++						+									Plasma/ serum	31
++			++								++		+			++	plasma	19
	+ or miRNA species-dependent	++ or miRNA species-dependent						+ or miRNA species-dependent									cell lines	29
		++								+							PBMC	16
		+									++						serum	16
	++	++			+				++					+++	+		Urinary exosomes	12

The number of "+" increases with the suitabiliy of the method in the same study.

The RNA extraction kits/methods are the following:

1. mirVana PARIS (Life Technologies)
2. mirVana (Life Technologies)
3. miRNeasy (Qiagen)
4. miRNeasy Serum/Plasma Kit (Qiagen)
5. QIAamp Circulating Nucleic Acids kit (Qiagen)
6. miRNeasy with RNeasy MinElute Cleanup kit (Qiagen)
7. mirPremier microRNA Isolation kit (Sigma)
8. High Pure miRNA isolation kit (Roche)
9. Trizol (LS)
10. Trizol (LS) + mirVana
11. Nucleospin miRNA kit (Macherey Nagel)
12. Nucleospin miRNA plasma kit (Macherey Nagel)
13. miRNA purification kit (Norgen Biotek)
14. Plasma/serum circulating RNA purification kit (Norgen Biotek)
15. Urine Exosome RNA Isolation kit (Norgen Biotek)
16. Mircury (Exiqon)
17. Mircury biofluids (Exiqon)
